# Linking disease epidemiology and livestock productivity: The case of bovine respiratory disease in France

**DOI:** 10.1371/journal.pone.0189090

**Published:** 2017-12-05

**Authors:** Alexis Delabouglise, Andrew James, Jean-François Valarcher, Sara Hagglünd, Didier Raboisson, Jonathan Rushton

**Affiliations:** 1 Veterinary Epidemiology, Economics and Public Health Group, Department of Production and Population Health, Royal Veterinary College, University of London, Hawkshead Lane, Hatfield, Hertfordshire, United Kingdom; 2 Veterinary Epidemiology and Economics Research Unit, University of Reading, Reading, Berkshire, United Kingdom; 3 Swedish University of Agricultural Sciences, Ruminant Medicine & Veterinary Epidemiology, Uppsala, Sweden; 4 IHAP, Université de Toulouse, INRA, ENVT, Toulouse, France; 5 Institute of Infection and Global Health, University of Liverpool, Liverpool, United Kingdom; The University of Melbourne, AUSTRALIA

## Abstract

Concerns are growing over the impact of livestock farming on environment and public health. The livestock industry is faced with the double constraint of limiting its use of natural resources and antimicrobials while ensuring its economic sustainability. In this context, reliable methods are needed to evaluate the effect of the prevention of endemic animal diseases on the productivity of livestock production systems. In this study, an epidemiological and productivity model was used to link changes in Bovine Respiratory Disease (BRD) incidence with the productivity of the beef and dairy cattle sectors in France. Cattle production parameters significantly affected by BRD were selected through literature review. Previous field study results and national cattle performance estimates were used to infer growth performances, mortality rates and carcass quality in the cattle affected and not affected by BRD. A steady-state deterministic herd production model was used to predict the productivity of the dairy and beef sector and their defined compartments (breeding-fattening, feedlot young bulls, and feedlot veal) in case of BRD incidence reduction by 20%, 50% or 100%. Results suggested that BRD should be controlled at a priority in beef breeding farms as eradication of BRD in beef calves would increase the whole beef sector’s productivity by 4.7–5.5% while eradication in other production stages would result in lower productivity gain in their respective sectors. However, the analysis performed at compartment level showed that, in both the beef and dairy sector, young bull and veal feedlot enterprises derive more economic benefits from BRD eradication for their own compartment (increase in productivity of 8.7–12.8% for beef young bulls) than the breeding farms (increase in productivity of 5.1–6% for beef calves), which may limit the investments in BRD control.

## Introduction

The rise of antimicrobial resistance as a major public health threat and growing concerns on the environmental impacts of the livestock industry have driven considerable attention to the issueof prevention and treatment of endemic livestock diseases [1]. The livestock industry is faced with the double constraint of limiting its use of natural resources (land use for feed production and grazing, water input) and antibiotic consumption while ensuring the economic sustainability of husbandry enterprises. For this reason, reliable methods are needed to evaluate the economic and environmental impact of prevention measures aimed at reducing the incidence of endemic livestock pathogens, in line with the societal need of improving animal welfare. In particular, linking levels of disease incidence with productivity (i.e. level of output produced with a given quantity of inputs) remains challenging.

Most studies tend to focus on visible production losses and additional expenditures in treatment, with few recording changes in herd structure or shifts in resource use. In reality these estimates are gross changes in the system rather than net estimates that require data on how inputs vary according to the production performance level. For example, losses caused by decreased average daily gain (ADG) of livestock due to diseases might be partly compensated by a decreased level of feed intake. On the other hand, a longer livestock rearing period might be required to reach a given standard slaughter weight, increasing the overall production cost. Through their effect on herd parameters such as mortality rate, age at maturity and fertility, endemic diseases indirectly affect the whole herd structure [2]. Measuring productivity changes allow a much more refined estimate of the economic impact of disease and health problems. Moreover most studies tend to focus on the impact of livestock diseases in specific production stages (e.g. breeding or fattening stage) and do not attempt to compare the relative effect of disease control in different compartments on the productivity of the whole system.

Respiratory diseases of cattle are a good example of this methodological gap. They are caused by a great diversity of pathogens infecting the lower and/upper respiratory tract of cattle, resulting in a clinical syndrome commonly named Bovine Respiratory Disease complex (BRD). BRD is a multifactorial disease. It has been frequently associated with infection by bovine respiratory syncytial virus (BRSV) and the bacteria *Mycoplasma bovis* but incriminated viruses also include bovine herpes virus type 1 (BHV-1), bovine coronavirus (BCoV), bovine parainfluenza 3 (BPIV-3), bovine adenovirus type 3 (BAdV-3), and bovine viral diarrhea virus (BVDV). Furthermore, bacterial agents such as *Mannheimia haemolytica*, *Pasteurella multocida* and *Histophilus somni* are isolated in most cases, in association with a primary infection by the abovementioned pathogens [3]. Environmental stressors are major drivers of the disease. The risk of BRD is greatest during or soon after the transportation of cattle [4]. Cattle exposed to a high concentration of microbes in the air, low bedding quality, and limited housing surface per individual are more susceptible to BRD [5]. The concentration of cattle in large herds and the lack of supervision of birth and colostrum feeding of calves by farmers also increase the risk of BRD [6, 7].

The economic importance of BRD has been frequently mentioned in the literature, and BRD has been the focus of farm-level economic evaluations [8–10]. Despite the demonstrated indirect impact of BRD on the herd breeding performances, these studies tend to only include visible farm production losses (deaths and reduced carcass quality due to BRD) and treatment costs. Besides, to our knowledge, no evaluation of the impact of BRD on a national cattle production system was ever attempted.

The present study was conducted to evaluate the economic impact of BRD in France through a modelling approach. Specifically, the study aimed at estimating the effect of BRD on the productivity of the two main cattle production sectors of France (the dairy sector and the beef suckling sector) and the gains in productivity that can be expected from an improved control of BRD. It also aimed at comparing the relative effect of BRD control in different compartments of the French cattle production system (i.e. breeding units and feedlots) on the sectors’ productivity.

France constitutes an interesting case study for three reasons: it comprises the largest cattle population of Europe [11] and its population structure is complex and diverse. It therefore constitutes a relevant case study to create a generic model which can be, then, applied to other European countries. In addition, respiratory diseases of cattle have been subject to many studies in this country as they are considered to be a major limitation to the performances of the French cattle industry [7, 12–16]. However, the scientific knowledge produced so far has not yet been valorised into a national scale economic evaluation.

## Study overview

### 1. Methodological framework of the study

**Fig 1:**
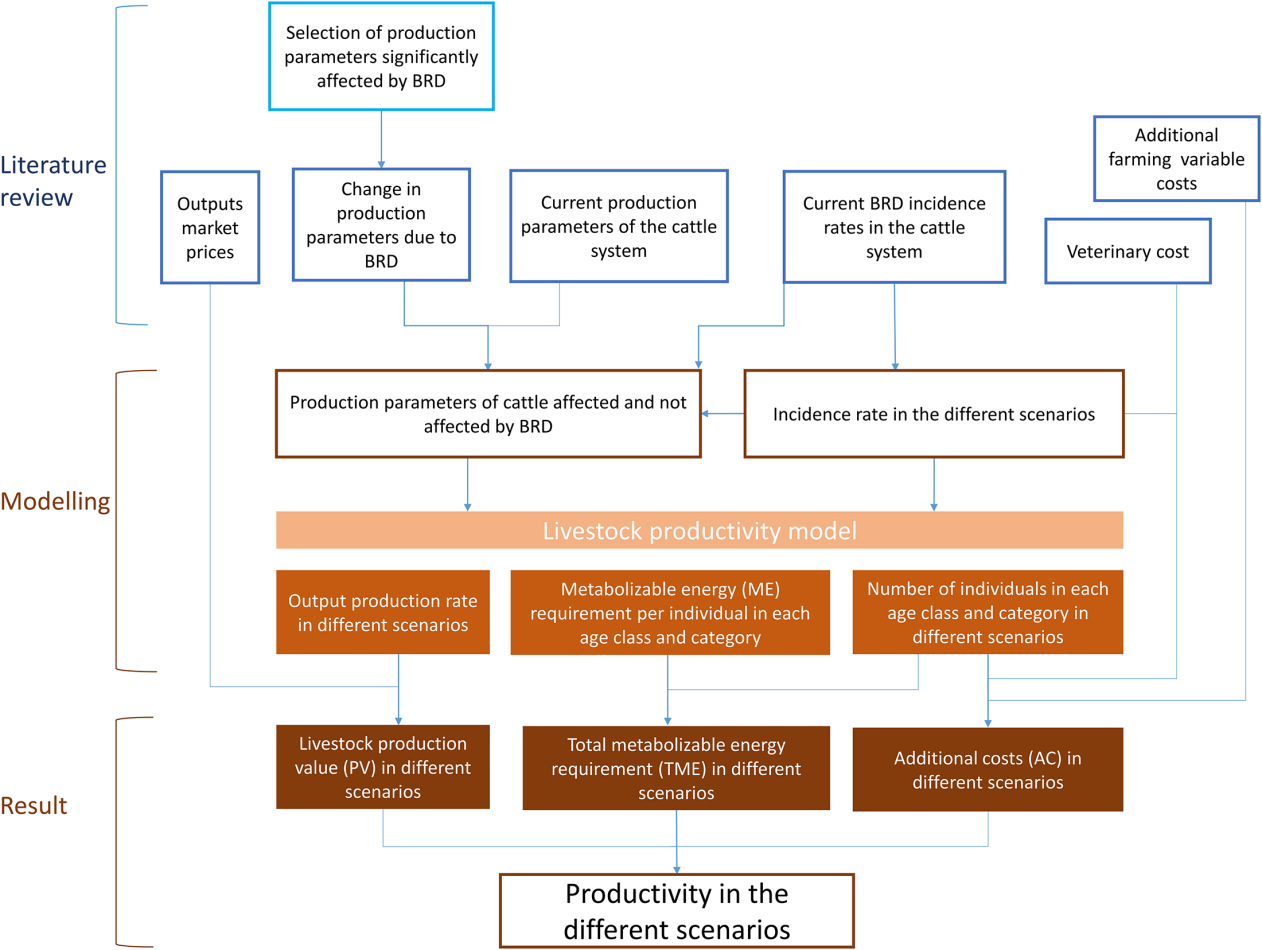
Methodological framework of the study. The productivity model was used to infer the production value (PV), the total metabolizable energy requirement (TME) and the additional costs (AC) of the considered livestock system under alternative scenarios corresponding to status quo and different levels of BRD incidence rate reduction. Input data were obtained from the literature.

The methodological framework of the study is illustrated in Fig 1. The productivity of the cattle production system under “status quo” scenario was compared with the productivity under alternative scenarios corresponding to different levels of BRD incidence rate reduction (namely 20%, 50% and 100%) in different compartments of the cattle production system.

Details of the productivity assessment are in **Material and Methods part 2.1**. The model used to estimate the productivity of the cattle production system is based on the Livestock Production Efficiency Calculator (LPEC) [17]. The productivity of the cattle industry, as measured by the model, is the ratio of the value of its production and the quantity of metabolizable energy (ME) it requires, the latter being a critical resource input to any livestock system and one that needs to be optimised in terms of environmental impact assessments as the energy source is a proxy for water and land use.

A literature review was performed beforehand to (i) identify the cattle production parameters significantly affected by BRD, (ii) quantify the effect of BRD on the selected cattle production parameters and (iii) estimate the current BRD incidence rates, production parameters, market prices of cattle products, variable farming costs (apart from feed) and veterinary costs associated with BRD cases in the French cattle production system (Fig 1). Details of the literature review are in **Material and Methods part 1**. Based on these data, production parameters of cattle affected and not affected by BRD during their production period were estimated, as described in **Material and Methods part 2.2.**

### 2. Structure of the French cattle production system

The French cattle production system is composed of two distinct sectors of comparable size: the beef suckling sector (hereafter referred as “Beef sector”, including 4.2 10^6^ cows (i.e. adult breeding females)) and the dairy sector (including 3.7 10^6^ cows) [18]. The two sectors use distinct breeds with specific breeding and growing performances which have been selected for the purpose of milk and meat production respectively.

**Fig 2:**
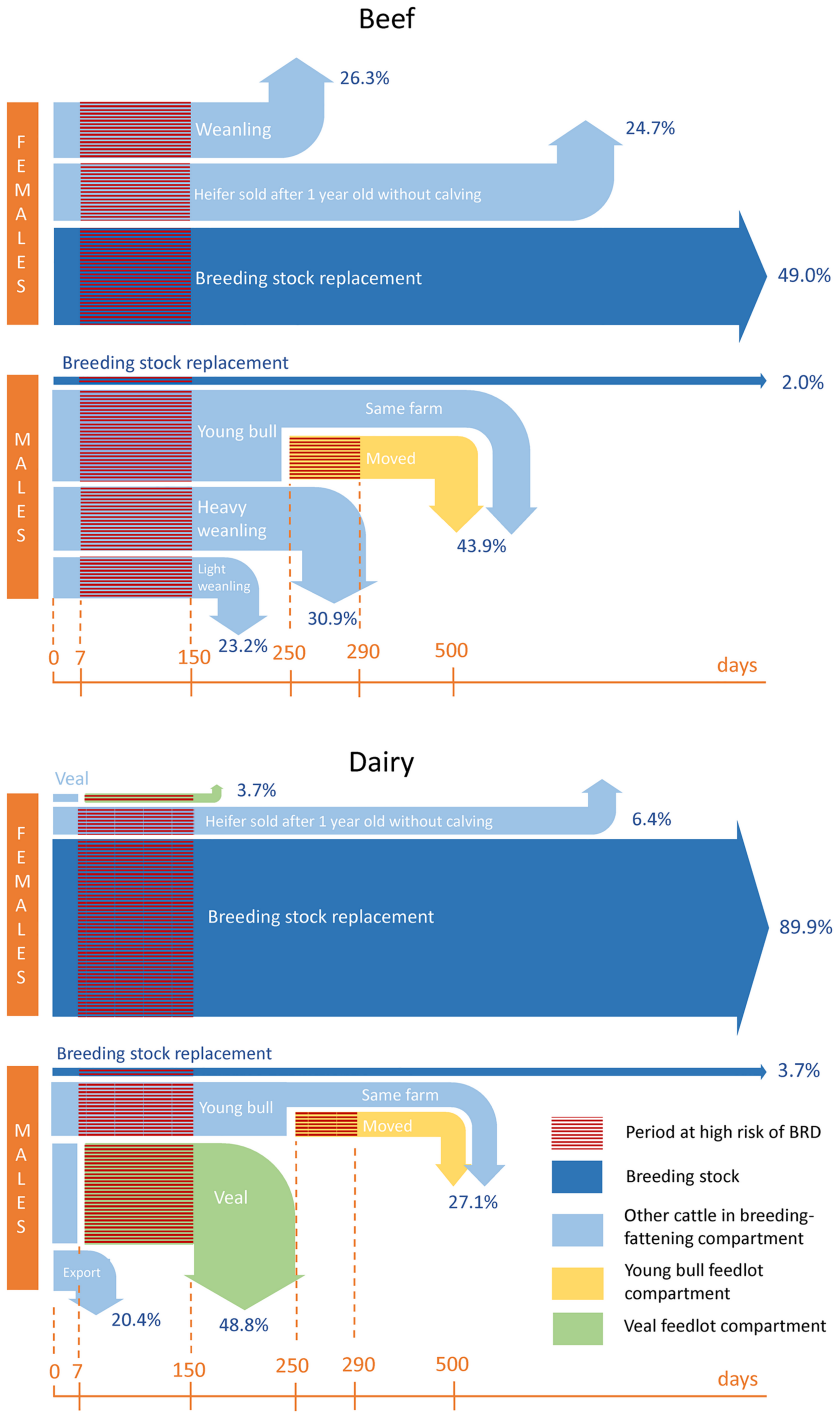
Representation of the French cattle value chain used in the study. Arrow sizes and percentages indicate the assumed proportion of calves used for the different types of purposes in the beef and the dairy sector and periods at risk of BRD. Arrow ends correspond to the approximate time of departure from the livestock system (slaughter or export). Corresponding ages are indicated on the bottom orange timeline. Source: Groupe Economie du Bétail Institut de l’Elevage. La production de viande bovine en France: qui produit quoi, comment et où? Paris: Institut de l’Elevage. 2011.

In the two sectors, a given proportion of newborn calves are used as breeding herd replacements, while others (hereafter referred as “surplus”) are used for a variety of other purposes, with different types of outputs and rearing periods, which are represented in Fig 2 along with their respective proportions. In the beef sector, males are either sold as young bulls between 1 and 2 years of age, after a period of fattening, or sold as weanlings (“broutards”). Weanlings are mainly exported to other countries for finishing. A distinction was made between weanlings sold early, right after weaning (“light weanlings”) or sold later, after a short pre-fattening period (“heavy weanlings”). Male dairy calves are either transferred to feedlot farms before weaning for veal meat production (at 1 week-1 month of age), transferred to feedlot farms after weaning for young bull production or exported to other countries (at 1 week-1 month of age) [19].

All male calves used for veal meat production and a large proportion of male calves used for young bull meat production are transferred to other farms for fattening. Therefore, each sector can be subdivided into “compartments” (Fig 2): (i) The breeding-fattening compartment, in both beef and dairy sectors, includes the breeding stock and breeding replacement stock and all the surplus cattle reared on-site until sale (for slaughter, export or additional fattening). (ii) The young bull feedlot compartment, in both beef and dairy sectors, includes male calves transferred to other farms after weaning and being fattened to produce young bull meat (42% of young bulls fattened in France) [19]. Weanlings are considered to be transported to young bull feedlots at 250 days of age. (iii) The veal feedlot compartment, only in the dairy sector, includes calves transferred to feedlot farms and fattened to produce veal meat. In the study, it was considered, for the sake of simplicity, that this transfer occurs at 1 week of age although in reality the age at transfer is comprised between 1 week and 1 month. Some minor types of surplus cattle were not included in the study: steers, exported female dairy calves and beef calves used for veal meat production. Together, these surplus represent less than 10% of the overall number of calves born per year in both beef and dairy sectors [19].

### 3. Production stages vulnerable to BRD

The scientific data produced on BRD in France and neighbouring countries suggest that BRD incidence rate is particularly high in calves in breeding farms between 7 days and 150 days of age [14], in male weaned calves moved to other farms for fattening (as opposed to weaned calves fattened in the farm where they are born), during the first few weeks after introduction in the feedlots [20], and in non-weaned calves being fattened in veal feedlot farms [21]. In consequence, effects of changes in BRD incidence rates in 5 specific at-risk production stages (2 at-risk stages in the beef sector, 3 at-risk stages in the dairy sector) on productivity were assessed (Fig 2): (i) Non-weaned beef calves from 7 days to 150 days old (in the breeding-fattening compartment of the beef sector), hereafter referred as “beef calves”; (ii) beef calves moved to a different farm for fattening, in the first 40 days after introduction in feedlot, i.e. from 250 to 290 days old (in the young bull feedlot compartment of the beef sector), hereafter referred as “beef young bulls”; (iii) Dairy calves from 7 days to 150 days old (in the breeding-fattening compartment of the dairy sector), hereafter referred as “dairy calves”; (iv) Veal calves from introduction in feedlot (at 7 days) to slaughter at about 6 months (in the veal feedlot compartment of the dairy sector), hereafter referred as “veal calves”; (v) Dairy young bulls moved to a different farm for fattening, in the first 40 days after introduction in feedlot, i.e. from 250 to 290 days old (in the young bull feedlot compartment of the dairy sector), hereafter referred as “dairy young bulls” (Fig 2).

As these at-risk production stages are in different compartments (breeding-fattening, young bull feedlot, veal feedlot) which correspond to different types of cattle farming enterprises, the effect of BRD incidence reduction in these production stages was assessed at the level of their sector (effect of BRD incidence reduction on the sector (beef or dairy) where it occurs) and their compartment (effect of BRD incidence reduction on the compartment where it occurs).

## Results

### 1. Literature review and model parameters

Many production parameters of cattle are potentially affected by BRD occurrence. Therefore, a first objective of the study was to select the effects of BRD to include in the model. A literature review was performed to identify cattle production parameters which were demonstrated to be significantly impacted by BRD. The results are summarized in Table 1, along with study references. According to the identified studies, BRD significantly increases the risk of premature death (mortality rate), decreases the ADG (i.e. average daily weight gain) and lowers the carcass quality of infected cattle. Besides, two studies demonstrated that a history of BRD occurrence during early years increases the risk of dystocia in breeding females (i.e. cows) at the time of calving. The reduction in ADG results either in lower weights at maturity or in delayed ages at maturity. However the effect of BRD on the fertility, survival after parturition, risk of abortion and milk production (quantity of milk and somatic cell count) of breeding females have not been clearly demonstrated or studies on these effects yielded contradictory results (Table 1). Therefore there is no consensus on whether these effects of BRD are true or not.

**Table 1:** Literature references on effects of BRD on cattle farms production performances.

Affected parameter	Stage of infection	Stage affected by change in production performances	Reference and Statistical significance of the observed effect	
			Significant	Not significant
Mortality rate	Dairy calf	Dairy calf	[22–24]	-
	Beef calf	Beef calf	[14, 23, 24]	-
	Veal calf	Veal calf	[21, 24]	-
	Feedlot cattle	Feedlot cattle	[20, 24–30]	-
ADG	Dairy calf	Dairy calf	[2, 31, 32]	[22]
	Beef calf	Beef calf	[33–35]	-
	Veal calf	Veal calf	[36, 37]	[38]
	Calf	Feedlot cattle	-	[39]
	Feedlot cattle		[20, 25, 27–29, 40–51]	-
Carcass quality	Feedlot cattle	Feedlot cattle	[20, 25, 28, 29, 43, 47–49, 52, 53]	-
	Veal calf	Veal calf	[37]	-
Age at first calving	Female calf	Heifer between weaning and calving	[2, 54–56]	[57, 58]
Risk of death before first calving			[2, 57, 59]	[58, 60]
Milk yield/ lactation	Female calf	Breeding female (cow)	[59]	[2, 56, 58, 61]
Somatic Cell Count			-	[56]
Survival after calving/Number of lactations			[56, 59]	[2, 62]
Parturition rate			-	[58, 59]
Risk of dystocia at calving			[2, 55]	-
Risk of abortion			-	[58]

Based on this literature review, a reduced number of BRD effects were chosen for inclusion in the model. They included the effect of BRD on risk of premature death (i.e. mortality risk), on ADG and, in the case of fattening cattle (veal calves and young bulls), on the risk of carcass downgrading. Quantified values of these effects were estimated through studies performed in France, except the change of ADG in dairy calves. As the latter was not estimated in the French context, the result of a study performed in United States was used. Estimates of BRD incidence rate or incidence risk were produced in previous studies done in France. The studies on BRD incidence and BRD-induced changes in production parameters used in the model are referenced in S1 Table along with their results. Probability density functions of disease incidence and effects of BRD on production parameters are displayed in Table 2.A (BRD incidence) and Table 2.B (Effect of BRD). Incidence rates were converted in incidence risks and conversely using the method explained in S1 Appendix.

**Table 2:** Biological parameters related with BRD and their assumed distribution used in the model. Study years and locations can be found in S1 Table.

A. BRD Incidence			
Stage of infection	Expression of the incidence	Study reference	Probability distribution
Beef calf 7–150 days	Incidence rate (per at-risk-day)	[14, 63, 64]	Normal *N*(*λ*, *σ*) *λ* = 1.89 .10^−3^ /day *σ* = 5.94 .10^−5^ /day
Dairy calf 15–75 days*	Cumulative incidence risk	[65]	Normal *N*(*p*, *σ*) *p* = 1.14 .10^−1^ *σ* = 1.5 .10^−2^
Veal calf 7 days to slaughter	Cumulative incidence risk	[21]	Normal *N*(*p*, *σ*) *p* = 2.7 .10^−1^ *σ* = 2.17 .10^−3^
Young bull 250–290 days	Cumulative incidence risk	[20, 25]	Normal *N*(*p*, *σ*) *p* = 1.94 .10^−1^ *σ* = 1.2 .10^−2^
B. Quantified effect of BRD on production parameters			
Stage of infection	Parameter changed	Study reference	Probability distribution
Beef calf 7–150 days	Mortality risk due to BRD in beef calves 7–150 days	[24]	Constant: 9.67%
	Difference of ADG in beef calves 7–150 days	[34, 35]	Normal *N*(*β*, *σ*) *β* = - 7.2 .10^−2^ kg/day *σ* = 1.17 .10^−2^ kg/day
Dairy calf 7–150 days	Mortality risk due to BRD in dairy calves 7–150 days	[24]	Constant: 3.40%
	Difference of ADG in dairy calves 7–150 days	[32]	Normal *N*(*β*, *σ*) *β* = - 5.9 .10^−2^ kg/day *σ* = 1.55 .10^−2^ kg/day
Veal calf 7 days to slaughter	Mortality risk due to BRD in veal calves 7 days—6 months	[24]	Constant: 2.90%
	Difference of ADG in veal calves 7 days—6 months	[37]	Normal *N*(*β*, *σ*) *β* = - 6.8 .10^−2^ kg/day *σ* = 8.86 .10^−3^ kg/day
	Difference of proportion of downgraded carcasses in veal calves at slaughter	[37]	Normal *N*(*α*, *σ*) *α* = 1.67 .10^−1^ *σ* = 1.26 .10^−2^
Young bull 250–290 days	Mortality risk due to BRD in young bulls 250–290 days	[24]	Constant: 8.77%
	Difference of ADG in young bulls 250–365 days	[25]	Normal *N*(*β*, *σ*) *β* = - 3.3 .10^−1^ kg/day *σ* = 9.53 .10^−3^ kg/day
	Difference of proportion of downgraded carcasses in young bulls at slaughter	[25]	Normal *N*(*α*, *σ*) *α* = 1.8 .10^−1^ *σ* = 4.92 .10^−2^

* The study only measured incidence risk in non-weaned dairy calves from 15 to approximately about 75 days old. Assuming an approximately constant incidence rate from 7 days until 150 days of age, the incidence risk was converted to a measure of incidence rate which was used to estimate the incidence risk over the full at-risk period (7–150 days)

Growth performance parameters are displayed in the S2 Table and breeding performance, milk production performances and feed metabolizability parameters are displayed in S3 Table. The National estimates of mortality rates in the different classes of ages in the two sectors were taken from [66]. Estimated veterinary costs associated with BRD cases were taken from [21]. 2015–2016 Market prices are displayed in the S4 Table. Estimated additional variable costs are displayed in S5 Table.

### 2. Model results: Effect of BRD incidence reduction on the demography and productivity of the French cattle production system

#### 2.1. Predicted effect of BRD on the age at maturity of breeding females and young bulls

**Fig 3:**
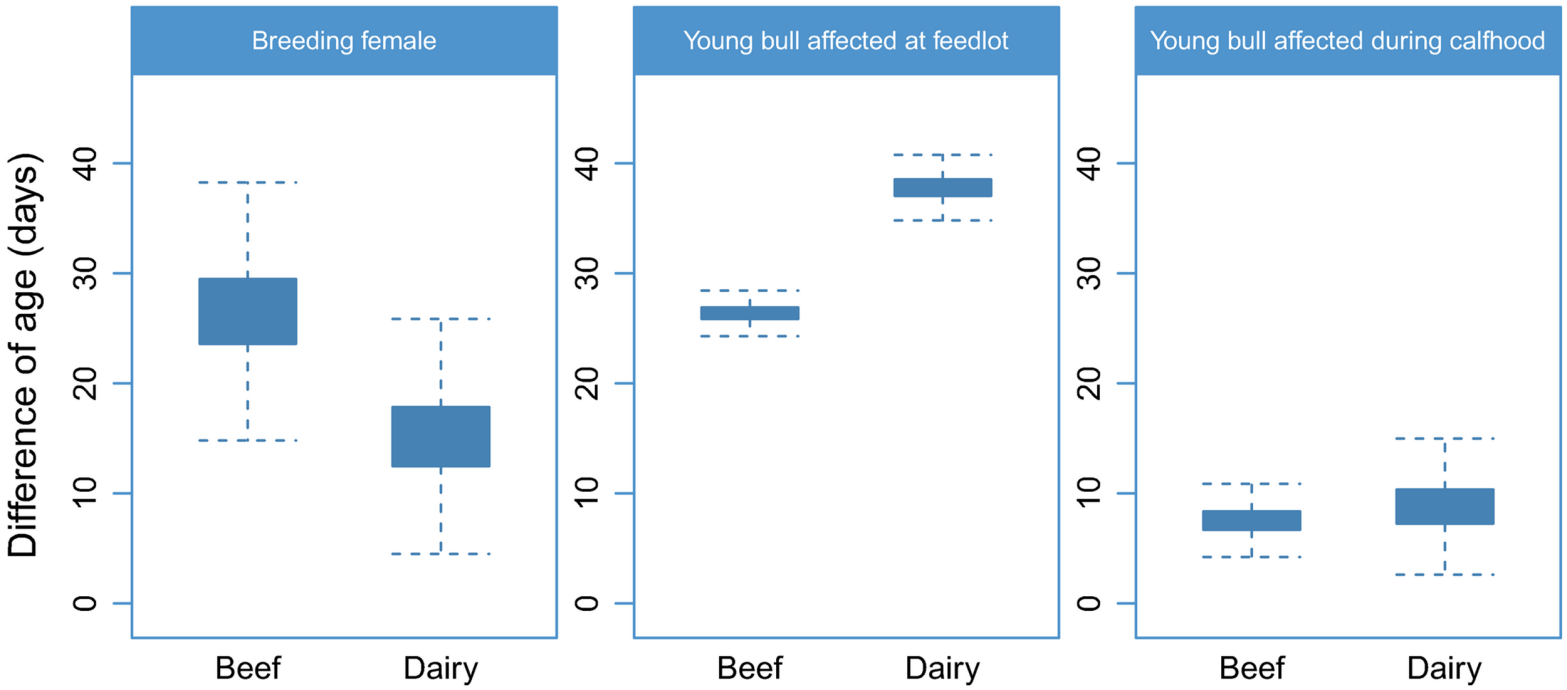
Box-and-whisker representation of predicted differences in age at maturity of breeding females and young bulls affected and not affected by BRD in the beef and dairy sector.

To illustrate the effect of the BRD-induced changes of ADG on the demographic structure of the cattle population, the differences of age at maturity between BRD affected and non-affected cattle predicted by the model are displayed in Fig 3. BRD occurrence during calfhood substantially delays the age at maturity in both males and females. Females used for breeding and affected by BRD during calfhood have their first calving 26.6 days (95% confidence interval (CI): 95%: 18–35.1) and 15.2 days (95% CI: 7.4–22.9) later in the beef and dairy sectors respectively. In male young bulls, BRD occurrence at feedlot has substantially more effect on the age at maturity than BRD occurrence in calfhood (26.4 days (95% CI: 24.9–27.9) and 7.5 days (95% CI: 5.1–9.9) respectively in the beef sector) (Fig 3).

#### 2.2. Predicted effect of BRD incidence reduction in calves on the demographic structure of the cattle population

According to model results, eradicating BRD in beef and dairy calves would have a substantial effect on the demographic structure of the female cattle population, as the reduction in calves’ mortality rate would allow a higher proportion of female calves to be used as surplus. In response to BRD eradication, the proportion of female calves used as surplus would increase by 1.3% (95% CI: 1.2–1.4) and 0.8% (95% CI: 0.6–1.0) in the beef and dairy sector respectively. In response to BRD incidence reduction by 50%, the proportion of female calves used as surplus would increase by 0.6% (95% CI: 0.6–0.6) and 0.4% (95% CI: 0.3–0.4) in the beef and dairy sector respectively.

#### 2.3. Predicted effect of BRD incidence reduction on the cattle system productivity

**Fig 4:**
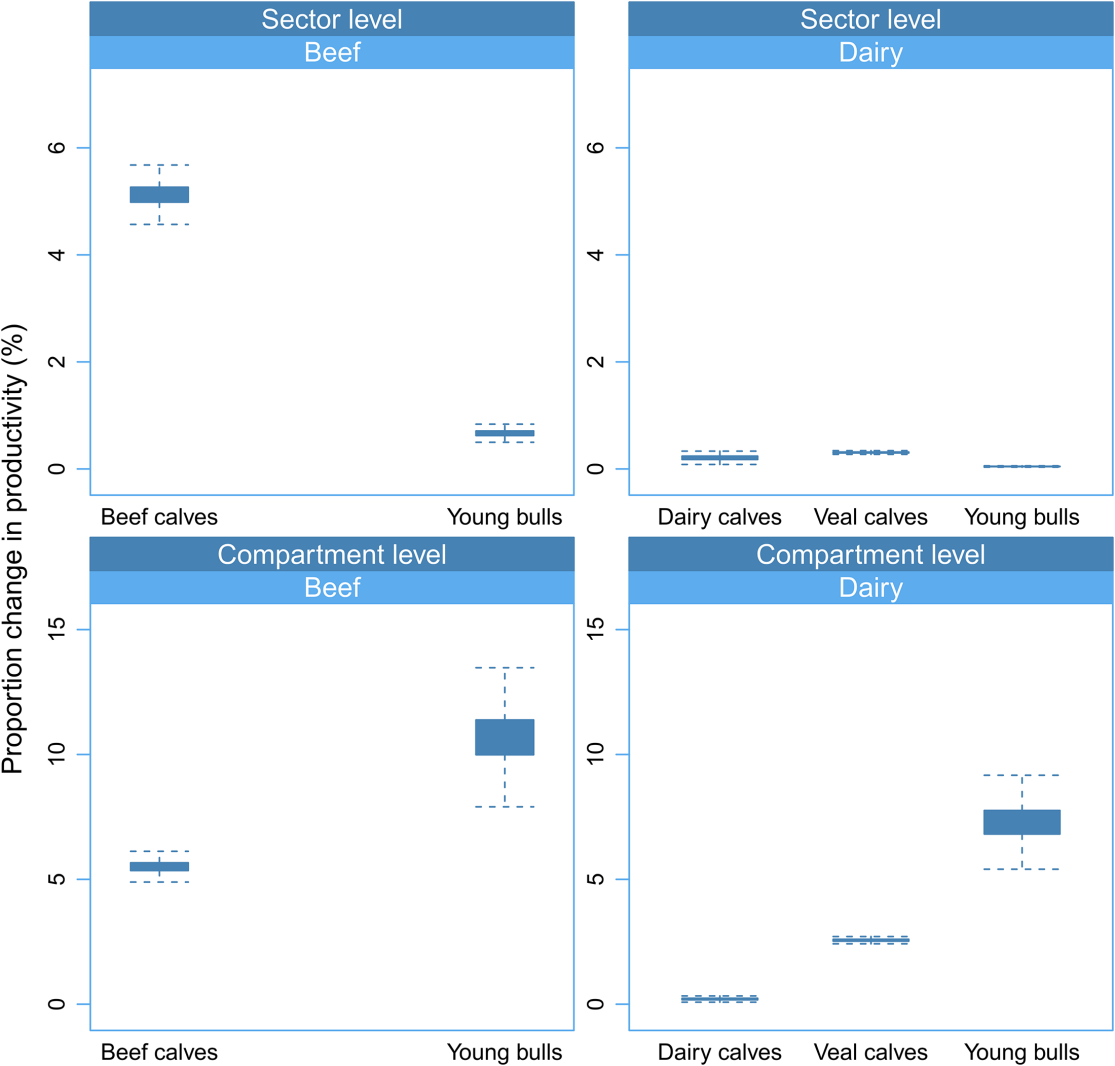
Ranges of variation in productivity of the French beef and dairy sectors and their specific compartments in response to BRD eradication in different production stages. Ranges are represented with box-and-whisker plots. Effects are differentiated according to sector and production stage where BRD is eradicated and level of analysis (sector or compartment).

Predicted changes in productivity resulting from BRD incidence reduction by 20%, 50% or 100% are displayed in Tables 3 and 4 and Fig 4. When considering the impact of BRD control at the level of the compartments where it occurs (breeding-fattening, young bull feedlots and veal feedlots), the highest gain of productivity would be obtained in the young bull feedlot compartment (Fig 4) with 10.7% (95% CI: 8.7–12.8%) and 7.3% (95% CI: 6–8.7%) increase in productivity in response to BRD eradication in the beef and dairy young bull feedlot compartment respectively (Table 3). However, predicted changes of productivity in response to BRD incidence reduction in young bulls are particularly sensitive to variation in market prices (Table 3). In both sectors, the lowest compartment-level gain in productivity would be in the breeding-fattening compartment, with 5.5% (95% CI: 5.1–6%) and 0.2% (95% CI: 0.1–0.3%) increase in productivity in response to BRD eradication in the beef and dairy breeding-fattening compartment respectively.

**Table 3:** Predicted changes in productivity of the breeding-fattening, young bull feedlot and veal feedlot compartments in response to BRD incidence reduction in their corresponding at-risk production stages. In each cell: Mean value (in bold type); between parenthesis: successively, 95% confidence interval with constant market values and 95% confidence interval with 5% variation in market values.

Sector	Compartment (production stage at risk)	Proportion incidence reduction		
		20%	50%	100%
Beef	Breeding-fattening beef (beef calves)	**1.1** (1–1.2; 1–1.2)	**2.7** (2.5–2.9; 2.4–3.1)	**5.5** (5.1–6; 4.8–6.3)
	Young bull feedlot	**2.2** (1.8–2.6; 1.6–3.1)	**5.4** (4.4–6.4; 3.9–7.7)	**10.7** (8.7–12.8; 7.8–15.3)
Dairy	Breeding-fattening (dairy calves)	**0** (0–0.1; 0–0.1)	**0.1** (0.1–0.1; 0.1–0.1)	**0.2** (0.1–0.3; 0.1–0.3)
	Veal feedlot	**0.5** (0.5–0.5; 0.4–0.6)	**1.3** (1.2–1.3; 1.1–1.4)	**2.6** (2.5–2.7; 2.2–2.9)
	Young bull feedlot	**1.5** (1.2–1.8; 1.2–1.9)	**3.7** (3–4.4; 2.9–4.7)	**7.3** (6–8.7; 5.7–9.3)

**Table 4:** Predicted changes in productivity of the French beef and dairy sectors in response to BRD incidence reduction in specific compartments. In each cell: Mean value (in bold type); between parenthesis: successively, 95% confidence interval with constant market values and 95% confidence interval with 5% variation in market values.

Sector	Compartment (production stage at risk)	Proportion incidence reduction			Financial benefit of BRD eradication (100% reduction) at national level (in million EUR/year)*
		20%	50%	100%	
Beef	Breeding-fattening beef (beef calves)	**1** (0.9–1.1; 0.9–1.1)	**2.5** (2.3–2.7; 2.2–2.9)	**5.1** (4.7–5.5; 4.5–5.8)	**95.5** (88.2–102.9; 87.8–103.1)
	Young bull feedlot	**0.1** (0.1–0.2; 0.1–0.2)	**0.3** (0.3–0.4; 0.3–0.4)	**0.7** (0.5–0.8; 0.5–0.8)	**12.4** (10.2–14.8; 9.9–15.5)
Dairy	Breeding-fattening (dairy calves)	**0** (0–0.1; 0–0.1)	**0.1** (0.1–0.1; 0.1–0.1)	**0.2** (0.1–0.3; 0.1–0.3)	**14.5** (8.6–21.4; 8.4–21.5)
	Veal feedlot	**0.1** (0.1–0.1; 0–0.1)	**0.1** (0.1–0.2; 0.1–0.2)	**0.3** (0.3–0.3; 0.3–0.4)	**21.4** (19.6–23.1; 18.3–24.5)
	Young bull feedlot	**0** (0–0; 0–0)	**0** (0–0; 0–0)	**0** (0–0.1; 0–0.1)	**3.2** (2.6–3.8; 2.5–4)

*Assuming constant Metabolizable Energy requirement of the sector

When considering the impact of BRD incidence reduction at the sector level (Table 4), the highest gain of productivity would be obtained in the beef sector, by reducing BRD incidence in beef calves in the breeding-fattening compartment. Eradicating BRD in this production stage would result in a 5.1% increase in the beef sector productivity (95% CI: 4.7–5.5%). In financial terms, assuming a constant ME requirement of the beef sector, this gain of productivity would represent an additional revenue of approximately 95.5 million EUR/year at national level. In comparison, BRD eradication in the dairy sector (in dairy calves, dairy young bulls or veal calves) would have a much lower effect on its productivity (Table 4).

### 3. Sensitivity analysis on market values

A sensitivity analysis was performed to determine how sector-level gains in productivity predicted by the model are likely to vary with changing market prices. The value of additional variable farming costs per cattle head have a strong positive effect on the predicted gain in productivity obtained from BRD incidence reduction in both the dairy and the beef sector. Culled beef breeding female price and milk price have a strong negative effect on the expected gains of productivity in, respectively, the beef sector and the dairy sector in response to BRD control in all stages. Expected gains of productivity in response to BRD incidence reduction in beef and dairy young bulls and veal calves are strongly affected by the corresponding standard and downgraded carcass prices (Table 5). The value of the veterinary cost of BRD mainly impacts the predicted gain in productivity from BRD incidence reduction in dairy calves, beef calves and veal calves.

**Table 5:** Results of the sensitivity analysis performed on market prices. Pearson correlation coefficients between model output (proportion change in sector productivity) and market prices. Only significant values (tested with Pearson correlation test, with 1% significance level) are displayed.

**Beef**				
**Production stage where BRD incidence is reduced**		**Beef calves**		**Beef young bull**
Heifer beef (carcass category*)	S			-0.05
	D	-0.04		-0.11
Young bull beef (carcass category*)	S	-0.18		+0.56
	D	-0.09		-0.46
Female beef weanling				-0.07
Light beef male weanling		-0.08		-0.07
Heavy beef male weanling		-0.13		-0.14
Beef breeding cattle		-0.48		-0.29
Additional farming cost		+0.83		+0.59
Veterinary cost of BRD		+0.17		+0.05
**Dairy**				
**Production stage where BRD incidence is reduced**		**Dairy calves**	**Veal calves**	**Dairy young bull**
Heifer dairy (carcass category*)	S	+0.05		-0.03
	D	+0.06		
Veal calf (carcass category*)	S	+0.03	+0.7	-0.04
	D		-0.42	
Young bull dairy (carcass category*)	S	+0.03		+0.74
	D			-0.36
Dairy breeding cattle		-0.05		
1 week old dairy calf				
Milk		-0.81	-0.53	-0.53
Additional farming cost	Dairy	+0.46	+0.2	+0.19
	Veal			
	Beef (young bull feedlots)	-0.12		
Veterinary cost of BRD		+0.32	+0.12	

* S: standard carcass, D: downgraded carcass

## Discussion

This study is the first one to estimate the overall impact of BRD at a national scale. The use of a productivity model allows integrating changes in the demographic structure of the livestock population and changes in input requirements as well as rate of output production. The used productivity model is based on an algorithm, the LPEC [17], which was originally designed for estimating the productivity of individual farms, but was successfully applied at national level in other case studies [67]. It has the advantage of not requiring estimating the quantity and unit cost of feed supplied to cattle. Instead, it predicts the quantity of ME supplied to the cattle population at equilibrium, given a set of production parameters, which makes it very convenient to apply in a large diversity of contexts. Besides, it allows accounting for all types of effects of diseases on production performances. As an example, if animals reach maturity after a standard rearing period (e.g. veal calves in this case) the reduction of ADG due to BRD affects the output value (i.e. carcass weight) while if animals reach maturity at a standard weight, the reduction of ADG delays the age at maturity (delayed first calving or delayed age at slaughter) which increases the number of “non-productive” animals (calves in their growing period) and, therefore, the ME requirements and variable costs per breeding females without modifying the output production rate and output value of the livestock system.

The study does not provide any estimation of the cost required to reduce the incidence or eradicate BRD. Eradication of BRD from cattle farms usually requires the mobilization of considerable resources from farmers or even proves to be impossible in practice. However, some studies suggested that BRD incidence can be significantly reduced at limited cost through improvements in herd management, including systematic check of the colostrum quality and colostrum intake of newborn calves, reduction of cattle group sizes and complete straw bedding of cattle [6, 7]. These farm-level control measures are difficult to cost but they can be assumed to represent moderate investments. Forecasting the decrease of BRD incidence resulting from improvements in farm biosecurity is a difficult task, but it can be assumed that reductions of 20% or 50% represent realistic objectives and such scenarios provide a reliable insight of the potential productivity gains to be expected from an improved control of respiratory pathogens of cattle.

At this stage, the specific impacts of individual pathogens were not differentiated. However, epidemiological studies conducted in the French beef sector showed BRSV and *Mannheimia haemolytica* are associated with most BRD cases [15]. Epidemiological data on French dairy sector are scarce. Some studies showed an important role of *Mycoplasma bovis* in veal calves’ respiratory diseases at feedlot [12, 68] but the prevalence of the bacteria in dairy breeding farms appears to be very low [13].

The results show that enhancing BRD control in beef breeding farms would substantially increase the productivity of the French cattle industry, reducing its environmental impact while satisfying consumers’ demand. Gains in productivity obtained through BRD control in other production stages (dairy calves in dairy breeding farms, young bull feedlots, veal farms) have a much lower impact on the productivity of their sectors. The lower effect of BRD incidence reduction in fattening young bulls on the productivity of the whole dairy and beef sectors is explained by the smaller proportion of fattening young bulls in the whole cattle population compared to non-weaned calves in both sectors. The lower economic impact of BRD incidence reduction in dairy calves compared with beef calves can be partly explained by the lower measured risk of mortality and ADG reduction in affected dairy calves compared to beef calves [24]. More importantly, most of the income of the dairy sector is derived from the milk produced by breeding females, and the income generated by surplus cattle is small in comparison. Therefore, a similar increase in the production of surplus cattle does not have the same effect on the overall productivity of the dairy and beef sector. It also explains why gains of productivity of the dairy sector are negatively correlated with milk prices and culled breeding cattle carcass prices.

In both sectors the compartment-level gain of productivity resulting from BRD incidence reduction is significantly higher in young bulls and veal feedlots than in the breeding-fattening compartment. This result highlights an important constraint to BRD control which is related to the cattle value chain structure. Indeed, recent studies demonstrated that the risk of BRD occurrence in veal feedlots depends on the level of immunoglobulin of veal calves at their introduction, i.e. the efficiency of passive immune transfer at birth [38], while the risk of BRD occurrence in young bulls feedlots depends on the level of seroconversion of newly introduced young bulls against the main respiratory pathogens [15]. In other words, BRD incidence in veal and young bull feedlots partly depends on prevention measures (colostrum feeding for veal calves, vaccination for young bulls) implemented in the breeding-fattening compartment while this later compartment derives lower economic benefits from BRD prevention. This unequal distribution of costs and benefits is likely to limit the investments in BRD control. A possible solution, in the case of young bull feedlots, would be to modulate prices of weaned calves sold to feedlot farms based on their vaccination status. However the vaccination history of weanlings can be difficult to trace, especially if their sale is mediated by many intermediate middlemen and the origin of the weanlings is not easily identifiable. An alternative solution is to introduce a vaccine at a sufficiently low price to motivate breeders to vaccinate their beef calves early in their life while providing a long lasting immunity, protecting calves until their fattening period.

The sensitivity analysis shows a dependence of the results on the market value of young bulls and veal carcasses, breeding cattle carcasses, milk and additional variable costs. As the market values of these components are likely to fluctuate in time, results of the model are expected to vary from one year to another. The strong positive correlation of the results with additional variable costs demonstrate the importance of accounting for changes in the demographic structure of the herd in response to better disease control: increase in calves’ ADG results in a reduction of the rearing period and, therefore, of the expenditures in feed and other daily farming costs. This correlation is not observed with additional variable cost of veal production, because the change in ADG in veal calves does not affect the duration of the rearing period.

The study was conducted in France, which has the largest cattle population in Europe. Results in other countries are expected to differ, depending on their epidemiological status for BRD (incidence rate in the different compartments) and the structure of their cattle production system. Based on the study results, it can be assumed that BRD mostly affects the productivity of cattle systems in which the beef sector and the young bull feedlots have a high economic importance and a high fraction of the calves are shipped to other farms for fattening.

One of the limits of the study is that it only accounts for the effect of clinical BRD, while most BRD cases are subclinical. Data on the effect of subclinical BRD on cattle production performances are much more limited. Subclinical BRD does not impact cattle mortality rate and is not associated with veterinary costs. However, the reduction in ADG and carcass quality resulting from subclinical BRD is still substantial, although lower than the ones resulting from clinical BRD [42, 69].

Some effects of BRD were not included in the model. It was demonstrated that occurrence of BRD in calfhood increases the risk of dystocia at calving [2, 55]. The economic cost of dystocia is difficult to evaluate, as it results both in additional time spent by farm workers in assisting calving and increased risk of health issues for breeding females and their newborn calves. Additional empirical data would be needed to properly address this specific effect of BRD.

The model does not consider any effect of BRD on the feed conversion ratio (FCR) of cattle. It is possible that the FCR of cattle affected by BRD increases, and, therefore, the relation between ADG and ME requirement (feed intake) might not be the same in affected and non-affected cattle. However, recent studies conducted on feedlot heifers in United States showed that increased FCR in sick animals during the infection phase is compensated later by a decrease in FCR, in comparison with non-infected cattle, during the compensatory growth phase. The authors of these studies concluded that the overall reduction in ADG can be largely, if not entirely, explained by a reduction in ME intake [28, 29].

The impact of BRD on the age at first calving of breeding females was indirectly included in the model, through the reduction of ADG during calfhood, which delays the age female reach the optimal weight for breeding. The predicted mean delay of 15.2 days in dairy females is consistent with the most recent results from empirical studies done in United States [2, 59]. Similarly, the predicted mean delay in age at slaughter of young bulls due to BRD at feedlot is similar the one measured in empirical studies in France [20].

The model required estimates of both incidence rate and incidence risk of BRD in the considered at-risk cattle populations. The method used to estimate one of these parameters from the other assumes independence between successive BRD affections, i.e. a BRD affection of one cattle does not reduce its risk of being affected at another time. This assumption cannot be verified. Nonetheless, a longitudinal epidemiological study of BRD in beef calves reported a substantial proportion of reoccurring BRD cases (around 10%) which shows that the risk of calves being affected more than once is significant [14].

The reliability of disease parameters used in the model strongly depends on the quality and reproducibility of the studies performed to estimate them. In most studies, definitions of BRD clinical cases are based on farmers’ decision to apply a clinical treatment. Criteria to judge whether animals need a medical intervention may vary between farm, and, most likely, between sectors, depending on the economic value of livestock. Case studies used to estimate biological parameters affected by BRD were performed on cattle of Prim’Holstein breed in the dairy sector and mostly Charolaise breed in the beef sector. Most dairy cattle in France are of Prim’Hostein breed while Charolaise breed accounts for more than 30% of cattle used in the beef sector [19]. It is not known whether the susceptibility and sensitivity of these two breeds to BRD affections significantly differs from other breeds.

## Conclusion

BRD control efforts should be focused in priority on beef breeding farms, as a decrease of BRD incidence in non-weaned beef calves would substantially enhance the productivity of the French cattle production system. However, at compartment level, in the beef and dairy sector, young bull and veal feedlot enterprises derive more economic benefits from BRD prevention than the breeding farms they purchase their cattle from, which may limit investments in BRD control.

## Material and methods

### 1. Literature review

A literature review was performed to identify quantified estimates of the effect of BRD occurrence on cattle production parameters. The literature review was conducted with the help of google scholar and PubMed using the following research terms: (i) “Bovine Respiratory Disease AND Performance”; (ii) “Bovine Respiratory Disease AND Production loss”; (iii) “Cattle AND pneumonia AND performance”; (iv) “Cattle AND pneumonia AND Production loss”. Besides, a specific research was conducted in the online records of the French veterinary theses (at: http://kentika.oniris-nantes.fr/) in order to identify studies conducted on BRD and published in French language. The used research term was “Respiratoire ET Bovin”.

Only studies providing quantitative estimates of the considered effect and assessing the significance of the effect with a statistical test where included in the review. A given effect was considered for inclusion in the model if its significance was demonstrated by at least half of the selected studies conducted on it.

Next, quantitative estimates of the selected effects were chosen for use in the model. These estimates were preferentially taken from studies conducted in France. When no studies done in France was identified, quantified estimates obtained in other countries where used. Similarly, estimates of BRD incidence were taken from surveys conducted in France.

Current production parameters of the French cattle system and veterinary costs linked to BRD were derived from results of national census or national cattle movements and slaughter databases. Product market prices and variable farming costs were found in online national market records. Sources of the data are detailed in S1–S5 Tables.

### 2. Modelling the effect of change in BRD incidence rate on the demography and productivity of the French cattle system

#### 2.1. The productivity model

The following definition of a livestock system productivity was used:

$$P=\frac{PV-AC}{TME}$$

*PV*: Value of all the products of the livestock system in one year (in monetary unit per breeding female per year).

*TME*: Total ME required by the livestock system (supplied by either forage, silage, concentrate feed or milk replacer) which is required to achieve the given level of performance (in Megajoule per breeding female per year)

*AC*: Additional variable costs (apart from feed) incurred by the livestock system (in monetary units per breeding female per year). It includes the expenditures in treatments of cattle affected by BRD, the purchase of animals (in the case of veal and young bull feedlot compartments), labour and other variable costs.

Note that a breeding female (i.e. cow) was defined as a female cattle during her reproductive period (i.e. from her first calving until her departure from the system).

Measures of the productivity of the considered cattle sectors with different levels of BRD incidence rate were determined using the same set of equations as in the LPEC algorithm [17]. The model is steady-state and deterministic, assuming a constant livestock population over time. Based on the mortality rates in the different age classes, the herd breeding performance parameters and the sex ratio of the breeding stock, the model determined the proportion of female and male calves used as breeding herd replacement in order to maintain a constant population. The rest of the newborn calves were distributed into different categories of destination (purposes), in proportions equal to the ones found in the literature (Fig 2). The model further divided these categories into two sub-categories, “affected by BRD during calfhood” and “not affected by BRD during calfhood” (“calfhood” referring either 7–150 days of age for calves kept in breeding farms until weaning or to the fattening period for veal calves). For young bulls moved to another farm for fattening, another sub-categorization was made between the ones affected and not affected by BRD during the 40 first days in feedlot. In each case, the proportion of calves in the sub-categories was directly determined from the estimated BRD incidence risk in each at-risk stage (S1 Appendix).

Each of these sub-categories of cattle, noted *i*, were attributed specific mortality rates, ADG, resulting weight and age at weaning and maturity, and output price which, in turn, determined their demographic weight in the herd (number of heads per breeding female, hereafter referred as *n*_*i*_) their ME requirement per unit of time, noted *me*_*i*_, their rate of output production *Ro*_*i*_ (i.e. quantity of output produced per breeding female per year) and output unit value *Vo*_*i*_.

Values of *PV*, *TME* and *AC* directly resulted from the set of equations:

$$PV=\sum_{i}^{n}\;{Ro}_{i}{Vo}_{i}$$

$$TME=\sum_{i}^{m}\;n_{i}{me}_{i}$$

$$AC=\sum_{i}^{m}\;n_{i}c_{i}+\omega_{i}c_{t}$$

With *m* the total number of sub-categories (determined by purpose and BRD status), *c*_*i*_ the additional variable cost per cattle head per unit of time in the sub-category *i*, *ω*_*i*_ the number of treatments of BRD cases administered in sub-category *i* per year and *c*_*t*_ the average veterinary cost of BRD cases treatment.

The demographic composition of each sub-category *n*_*i*_ was estimated using the same method as the LPEC algorithm [17]. The method is described in S2 Appendix.

The formulas used to determine ME requirements of each subcategory were the same as the ones used by the LPEC algorithm [17] and were supplied by the National Research Council [70]. Note that breeding females were attributed specific ME requirements determined by their breeding performances (parturition rate, milk production, weight loss in early lactation, weight at maturity and weight at culling), and an output production rate determined by their culling rate and milk production.

ME requirements were calculated differently in the beef and dairy sectors. In the beef sector it was assumed that breeding females were never milked and the non-weaned calves fed entirely from suckling their mothers. Therefore, the milk produced by breeding females was directly determined by the ME requirements of the non-weaned calves and these ME requirements were not included in the *TME*. In the dairy sector, breeding females were assumed to be entirely milked and the milk produced was either sold for human consumption or for feeding non weaned calves. The milk used to feed non weaned calves was considered to be entirely purchased (as raw milk or milk replacer). Therefore, the totality of the milk produced by breeding females was considered as an output of the livestock system while ME requirements of non-weaned calves were included in the *TME*.

The formula used to estimate the ME required by breeding females to produce 1 kg of milk was supplied by the National Research Council [71]:

$$4.184(0.192+0.0929\mu_{f}+0.0563\mu_{p})$$

*μ*_*f*_ and *μ*_*p*_ being the concentration of fat and protein in the milk respectively.

#### 2.2. Production parameters in “BRD affected” and “non BRD affected” cattle categories

All values were based on current estimations of incidence rates *λ* and incidence risks *p* of BRD in the cattle population of France during the defined at-risk periods.

The basic mortality rate *τ*_0_ (i.e. mortality due to anything but BRD) was determined using the following equation:

$$\tau_{0}=\tau -\kappa \lambda$$

With *τ* the current mortality rate in the population over the considered period, *κ* the mortality risk due to BRD and *λ* the current BRD incidence rate in the population.

The following formula was used to estimate the mortality rate *τ*_1_* of cattle affected by BRD in the course of the considered at-risk period in a given scenario *:

$${\tau_{1}}^{*}=\frac{1}{t}\text{ln}\;\left(\frac{p^{*}}{e^{-(\tau_{0}+\kappa \lambda^{*})t}-(1-p^{*})e^{-\tau_{0}t}}\right)$$

With *t* the duration of the at-risk period and λ* and p* the incidence rate and incidence risk in the scenario * respectively. The mathematical bases of this formula are explained in S3 Appendix.

The distribution of ADG in the cattle population was considered to be a mixture of two normal distributions *N*(*δ*_0_, *σ*_0_) and *N*(*δ*_1_, *σ*_1_) corresponding to cattle not affected and affected by BRD during the at-risk period respectively. Therefore, the mean ADG *δ* of the population during the considered critical period was considered to be *δ* = *pδ*_1_ + (1 − *p*)*δ*_0_ and *δ*_1_ − *δ*_0_ = *β*, *β* being the estimated regression coefficient of BRD status on ADG. Therefore mean ADGs of cattle of the considered class of age (beef and dairy calves, veal calves and young bulls) according to their BRD status (respectively *δ*_1_ and *δ*_0_) were inferred from *δ*, *p* and *β*:

$$\delta_{0}=\delta -p\beta$$

$$\delta_{1}=\delta +(1-p)\beta$$

The duration of the rearing period of veal calves (time from birth to sale for slaughter) was considered to be independent on their ADG, as veal calves were assumed to be farmed in all-in-all-out systems. However, the final weight reached by veal calves at sale time was considered to depend on their ADG. Therefore the mean total weight gains reached by veal calves, not affected or affected by BRD, noted *w*_0_ and *w*_1_ respectively, over period *t* were considered to be:

$$w_{0}=t\delta_{0}$$

$$w_{1}=t\delta_{1}$$

On the other hand, the weight at weaning and maturity of breeding herd replacement cattle, weanlings, heifers sold after 1 year, and young bulls were considered to be independent of the ADG. Therefore the mean rearing period duration needed for these categories of cattle not affected and affected by BRD, to reach a constant total weight gain *w*, noted *t*_0_ and *t*_1_ respectively, were considered as:

$$t_{0}=w\;(\frac{1}{\delta_{0}}+\frac{{\sigma_{0}}^{2}}{{\delta_{0}}^{3}})$$

$$t_{1}=w\;(\frac{1}{\delta_{1}}+\frac{{\sigma_{1}}^{2}}{{\delta_{1}}^{3}})$$

*σ*_0_ and *σ*_1_ being the true standard deviations of the ADGs of cattle not affected and affected by BRD, respectively. *σ*_0_ and *σ*_1_ were assumed to be equal to the current standard deviation of ADG in the cattle population, which is approximately equal to 0.2 kg/day [25, 35].

Risks of downgrading of carcasses of veal calves or young bulls affected and not affected by BRD (respectively noted *γ*_0_ and *γ*_1_) were calculated as:

$$\gamma_{0}=\gamma -\alpha p$$

$$\gamma_{1}=\gamma +\alpha (1-p)$$

With *γ* the current proportion of downgraded carcasses in the population and *α* the estimated difference between downgrading risk of affected and unaffected cattle.

Under a given scenario with BRD incidence rate λ* in a defined at-risk period, the number of treatments ω* administered for BRD affection per breeding female per year was considered to be:

$$\omega^{*}=n\lambda^{*}$$

With *n* the number of cattle in the at-risk period per breeding female estimated by the productivity model.

### 3. Probability distribution of model outputs and sensitivity analysis on disease parameters and prices

Estimates of BRD incidence rate and effects of BRD on ADG and carcass quality were obtained from previous cross-sectional or longitudinal studies performed on samples of the cattle population. The precision of the estimates being limited by the used sample sizes, the uncertainty on these variables was addressed through a stochastic approach. The probability density functions of model parameters (incidence rate or incidence risk, treatment cost, BRD-induced change of ADG, BRD-induced change of risk of carcass downgrading) were determined from their sample estimate and standard error, t-test value, or p value, depending on the information supplied in the study reference. Ten thousand values of the abovementioned parameters were sampled from their modelled probability distribution. The sampling followed a random Latin Hypercube Sampling approach, using the R package “lhs” [72]. For each iteration, corresponding changes in productivity in response to given changes in BRD incidence rates were estimated. The variability of market values (livestock products prices and additional farming costs) was also accounted for using the same method. As the true probability distributions of market values are unknown, uniform probability distributions bounded by minimum and maximum values, corresponding to a decrease or increase of 5% of these market values respectively, were simulated. Current production parameters of the French cattle system were obtained from results of national census or estimates made on large cattle populations. Therefore, it was considered that the uncertainty on these parameters is weak and their probability distribution was not modelled.

Besides, a sensitivity analysis was performed to assess the dependency of the model results to market values (cattle products’ market prices and variable costs). The relative effect of each market value was estimated from the Pearson product correlation coefficient between model outputs (the proportion change of the sector productivity) and sampled model inputs (the considered market value).

### 4. Computational material

All computational analysis and graphical representation of results were performed using the version 3.2.0 of R [73].

## Supporting information

S1 Appendix Relation between incidence rate and incidence risk. (DOCX)

S2 Appendix Demographic weight of each sub-category and classes of age. (DOCX)

S3 Appendix Rates of mortality in the “non-affected” and “affected” cattle categories. (DOCX)

S1 Table Estimations of biological parameters used in the study. (DOCX)

S2 Table Fixed parameters used in the productivity model: average weights and ages at birth, weaning and maturity in the different cattle categories. (DOCX)

S3 Table Fixed parameters used in the productivity model: breeding milk production performances and nutritional parameters. (DOCX)

S4 Table Market prices used in the study (2015–2016 prices). (DOCX)

S5 Table Additional variable farming costs (per cattle-year). (DOCX)
