# Supplementary material for: Linking disease epidemiology and livestock productivity: The case of bovine respiratory disease in France
Source: PLoS One. 2017 Dec 5;12(12):e0189090. doi: 10.1371/journal.pone.0189090 (PMC5716546; doi:10.1371/journal.pone.0189090)
Supplement: S1 Appendix — (DOCX) [file pone.0189090.s001.docx]

**S1 Appendix. Conversion of incidence rate into incidence risk**

Values of BRD Incidence rates $\lambda$ were converted into BRD incidence risks $p$ (the risk of being affected by BRD over the at-risk period of duration $t$) using the following formula:

$$p=\frac{\lambda}{\tau_{0}+\lambda}\left(1-e^{-\left(\tau_{0}+\lambda\right)t}\right)$$

With $\tau_{0}$ the basic mortality rate (due to any cause except BRD) over the considered at-risk period. $\tau_{0}$ was determined using the following equation:

$$\tau_{0}=\tau-\kappa\lambda$$

With$\tau$ the current mortality rate observed in the population over the considered period and $\kappa$ the mortality risk due to BRD.

*Demonstration:*

Considering a simple population of cattle of similar age divided in two compartments:

$S$: cattle having never been infected during the at-risk period

$I$: cattle having been infected at least once during the at-risk period

At the beginning of the at-risk period, all cattle are in compartment$S$.

The evolution of the number of cattle in the compartment $S$ during the at-risk period is:

$$\frac{dS}{dt}=-(\lambda+\tau_{0}).S$$

Therefore, the proportion of individuals of compartment $S$, at time $t$, over the number of initial individuals is:

$S\left(t\right)=e^{-(\lambda+\tau_{0})t}$ and $S\left(0\right)=1$

The incidence risk (i.e. proportion of an initial population of individuals being infected at least once over an at-risk period $T$) is:

$$p=\int_{0}^{T} \lambda.S(t).dt=\int_{0}^{T} \lambda.e^{-(\lambda+\tau_{0})t}.dt=\frac{\lambda}{\lambda+\tau_{0}}\left(1-e^{-(\lambda+\tau_{0})T}\right)$$
