# Supplementary material for: Linking disease epidemiology and livestock productivity: The case of bovine respiratory disease in France
Source: PLoS One. 2017 Dec 5;12(12):e0189090. doi: 10.1371/journal.pone.0189090 (PMC5716546; doi:10.1371/journal.pone.0189090)
Supplement: S2 Appendix — (DOCX) [file pone.0189090.s002.docx]

**S2 Appendix. Demographic weight of each sub-category and classes of age**

The demographic weight of each sub-category of the cattle population was estimated using the same method as the LPEC algorithm. In each subcategory, the number of cattle of specific age $t_{i}$ produced per year per breeding female $k_{i}$ was calculated as:

$$k_{1}=k_{0}e^{-\tau_{0-1}t_{1}}$$

and

$\forall i\geq1$, $k_{i+1}=k_{i}e^{-\tau_{i-i+1}(t_{i+1}-t_{i})}$

With:

$k_{0}$ the number of newborn calves produced per breeding female per year which were in the considered subcategory (determined by the breeding females’ parturition rate, survival rate in the first day, proportion of calves in the category of use and BRD incidence risk).

$\tau_{0-1}$ the mortality rate between birth and age $t_{1}$

$\tau_{i-i+1}$ the mortality rate between ages $t_{i}$ and $t_{i+1}$

In each subcategory, the number of cattle comprised between ages $t_{i}$ and $t_{i+1}$ (noted$n_{i-i+1}$) was calculated as:

$$n_{i-i+1}=\frac{k_{i}}{\tau_{i-i+1}}\left(1-e^{-\tau_{i-i+1}(t_{i+1}-t_{i})}\right)$$
