# Supplementary material for: Linking disease epidemiology and livestock productivity: The case of bovine respiratory disease in France
Source: PLoS One. 2017 Dec 5;12(12):e0189090. doi: 10.1371/journal.pone.0189090 (PMC5716546; doi:10.1371/journal.pone.0189090)
Supplement: S3 Appendix — (DOCX) [file pone.0189090.s003.docx]

**S3 Appendix. Rates of mortality in the “non-affected” and “affected” cattle categories**

An important assumption is that the risk of BRD of a given cattle at any time is independent of his past infections. The rate of mortality in the “non-affected” category $\tau_{0}$ (cattle which are not affected by BRD along the period *t*) is:

$$\tau_{0}=\tau-\kappa.\lambda$$

With $\tau$ the mortality rate observed in the population over the considered period and $\kappa$ the mortality risk due to BRD.

In order to determine the rate of mortality $\tau_{1}*$ in the “affected” category (cattle affected by BRD at least once over the period *t*) in a given scenario *, the following expression of the proportion of surviving animals over the period $t$ can be used:

$$e^{-\left(\tau_{0}+\kappa\lambda*\right)t}=p.e^{-\tau_{1}*t}+(1-p).e^{-\tau_{0}t}$$

With $\lambda*$and $p*$ the incidence rate and incidence risk in the scenario * respectively. Therefore:

$$\tau_{1}*=\frac{1}{t}Ln\left(\frac{p*}{e^{-\left(\tau_{0}+\kappa\lambda*\right)t}-(1-p*)e^{-\tau_{0}t}}\right)$$
