# Supplementary material for: Linking disease epidemiology and livestock productivity: The case of bovine respiratory disease in France
Source: PLoS One. 2017 Dec 5;12(12):e0189090. doi: 10.1371/journal.pone.0189090 (PMC5716546; doi:10.1371/journal.pone.0189090)
Supplement: S1 Table — (DOCX) [file pone.0189090.s004.docx]

**S1 Table. Estimations of biological parameters used in the study.**

| 1. BRD Incidence   N: denominator (number of cattle included in the study or number of days-at-risk)  n: numerator (number of cattle affected by BRD or number of sick days)  SD: standard deviation | | | |
| --- | --- | --- | --- |
| Stage of infection | Expression of the incidence | Study reference, location and year | Study results |
| Beef calf 7-150 days | Incidence rate (per day) | 1-3  France, Pays de la Loire, 1999-2000 | 1.89.10^-3^ /day-at-risk-calf  n = 1010  N = 534504 |
| Dairy calf 15-75 days | Incidence risk (proportion of cattle infected over the period) | 4  Pays de la Loire, 1995-1996 | average 11.4% calves affected per farm, calculated on 256 dairy farms  SD: 24% |
| Veal calf 7 days to slaughter | Incidence risk  (proportion of cattle infected over the period) | 5  France, Northwest, 2005-2006 | 27% calves individually treated  N=42000 |
| Young bull in the 40 days after introduction in feedlot | Incidence risk  (proportion of cattle infected over the period) | 6, 7  France, Pays de la Loire, 2005-2006 | 19.4% affected  n = 210  N = 1083 |
| 1. Correlation between BRD incidence and production parameters   N: number of cattle included in the study  n: number of cattle in each category (affected or not affected by BRD)  p: p-value of the used test  SD: standard deviation of the estimated parameter | | | |
| Production stage | Parameter changed | Reference, location and year of study | Study results |
| Beef calf 7-150 days | Mortality risk in affected beef calves | 8  France, nationwide survey, 2001 | 9.67% |
|  | ADG in beef calves 7-150 days | 9, 10  France, Pays de la Loire, 1999-2000 | N = 3516  Linear regression:  BRD at 0-45 days of age:  -0.103 kg/day  (p = 0.0001, n =193)  BRD at 45-90 days of age:  -0.062 kg/day  (p = 0.0001, n = 215)  BRD at 90-150 days of age:  -0.049 kg/day  (p = 0.0042, n = 175) |
| Dairy calf 7-150 days | Mortality risk in affected dairy calves | 8  France, nationwide survey, 2001 | 3.40% |
|  | ADG in dairy calves 7-150 days | 11  USA, 1997 | N = 1972  n = 414 affected by BRD  Linear regression: - 0.0105 g/day for each day of treatment (p = 0.0001) with an average 5.63 days of treatment (SD : 5.33) |
| Veal calf 7 days to slaughter | Mortality risk in affected veal calves | 8  France, nationwide survey, 2001 | 2.90% |
|  | ADG in veal calves 7days – slaughter | 12  France, Bretagne and Pays de la Loire, 2007-2008 | N=1839  Difference: -0.068 kg/day  Non affected young bulls  (n = 964):  ADG = 1.047 kg/day  SD = 0.187 kg/day  Affected young bulls (n = 875):  ADG = 0.979 kg/day  SD = 0.192 kg/day |
|  | Carcass quality in veal calves at slaughter | 12  France, Bretagne and Pays de la Loire, 2007-2008 | Non affected young bulls : n = 964  Affected young bulls: n = 875  Difference in proportion of under-rated carcasses: 16.7 % |
| Young bull 250-290 days | Mortality risk in affected young bulls | 8  France, nationwide survey 2001 | 8.77% |
|  | ADG in young bulls 250-365 days | 7  France, Pays de la Loire, 2005-2006 | N = 190  Difference: -0.33 kg/day  Non affected young bulls  (n = 122):  ADG = 1.121 kg/day  SD = 0.060 kg/day  Affected young bulls (n = 68):  ADG = 0.791 kg/day  SD = 0.064 kg/day |
|  | Carcass quality in young bulls at slaughter | 7  France, Pays de la Loire, 2005-2006 | Non affected young bulls:  n = 122  Affected young bulls: n = 68  Difference of proportion of under-rated carcasses:  18.0 % |

**References**

1. Assié S, Bouet JM, Seegers H, Quillet JM. Economic impact related to respiratory disorders of non-weaned calves in Charolais cow-calf farms of Pays de la Loire (France). Renc Rech Ruminants. 2001;8:145-8.

2. Assié S. Incidence, impact économique et facteurs de risque des troubles respiratoires des veaux charolais non sevrés en système d'élevage allaitant. Nantes, France: Ecole Nationale Vétérinaire de Nantes; 2004. Available from: http://kentika.oniris-nantes.fr/Main.htm?context=2.

3. Assie S, Seegers H, Beaudeau F. Incidence of respiratory disorders during housing in non-weaned Charolais calves in cow-calf farms of Pays de la Loire (Western France). Prev Vet Med. 2004;63(3-4):271-82.

4. Rio O. Fréquence et Risques de Mortalité et Troubles de Santé des Veaux en Elevage Laitier: Ecole Nationale Vétérinaire de Nantes; 1999. Available from: http://kentika.oniris-nantes.fr/Main.htm?context=2.

5. Martineau C, Bertrand G, Kergoulay P. Indicateurs Zootechniques et Sanitaires. Veaux de boucherie. Le Rheu, France: Institut de l'Elevage, GIE Lait-Viande de Bretagne, la Chambre Régionale d'Agriculture de Bretagne. 2007.

6. Bareille N, Seegers H, Denis G, Quillet JM, Assié S. Impact of respiratory disorders in young bulls during their fattening period on performance and profitability. Renc Rech Ruminants. 2008;15.

7. Joly M. Incidence, effet sur les performances et impact économique des troubles de santé des jeunes bovins en atelier d'engraissement en Pays de la Loire. Nantes, France: Ecole Nationale Vétérinaire de Nantes; 2007. Available from: http://kentika.oniris-nantes.fr/Main.htm?context=2.

8. Gay E, Barnouin J. A nation-wide epidemiological study of acute bovine respiratory disease in France. Prev Vet Med. 2009;89(3-4):265-71.

9. Delobel L. Maladies Respiratoires des Veaux Charolais Non Sevrés en Pays de la Loire: Relations avec la Croissance. Nantes, France: Ecole Nationale Vétérinaire de Nantes; 2003. Available from: http://kentika.oniris-nantes.fr/Main.htm?context=2.

10. Assié S, Delobel L, Seegers H, Beaudeau F. Relationships between growth rates and occurrence of respiratory disorders of nonweaned calves in Charolais cow-calf farms of Pays de la Loire (France). Renc Rech Ruminants. 2003;10:293-6.

11. Donovan GA, Dohoo IR, Montgomery DM, Bennett FL. Calf and disease factors affecting growth in female Holstein calves in Florida, USA. Prev Vet Med. 1998;33:1-10.

12. Pesneau E. Incidence et Impact sur les Performances de Croissance et d'Abattage des Troubles Respiratoires des Veaux de Boucherie. Nantes, France: Ecole Nationale Vétérinaire de Nantes; 2008. Available from: http://kentika.oniris-nantes.fr/Main.htm?context=2.
