# Supplementary material for: Linking disease epidemiology and livestock productivity: The case of bovine respiratory disease in France
Source: PLoS One. 2017 Dec 5;12(12):e0189090. doi: 10.1371/journal.pone.0189090 (PMC5716546; doi:10.1371/journal.pone.0189090)
Supplement: S2 Table — (DOCX) [file pone.0189090.s005.docx]

**S2 Table. Fixed parameters used in the productivity model: average weights and ages at birth, weaning and maturity in the different cattle categories.**

| Beef sector | | | | | | | |
| --- | --- | --- | --- | --- | --- | --- | --- |
|  | | Weight at birth (kg) | Weight at weaning  (kg) | Weight at maturity (kg)* | Average daily gain (kg/day) birth-150 days | Average daily gain (kg/day)  150-250 days | Average daily gain (kg/day)  >250 days |
| Female | Breeding herd replacement | 45 | 300 | 675** | 1.04 | 1.11 | 0.49 |
|  | Weanling | 45 | 300 | 325 | 1.04 | 1.11 | 0.49 |
|  | Heifer sold after 1 year old without calving | 45 | 300 | 688 | 1.04 | 1.11 | 0.49 |
| Male | Breeding herd replacement | 48 | 325 | 1000 | 1.14 | 1.34 | 1.49 |
|  | Light weanling | 48 | 300 | 300 | 1.14 | 1.34 | 1.49 |
|  | Heavy weanling | 48 | 325 | 420 | 1.14 | 1.34 | 1.49 |
|  | Young bull | 48 | 325 | 755 | 1.14 | 1.34 | 1.49 |

| Dairy sector (without veal production) | | | | | | | |
| --- | --- | --- | --- | --- | --- | --- | --- |
|  | | Weight at birth (kg) | Weight at weaning  (kg) | Weight at maturity (kg) | Average daily gain (kg/day) birth-150 days | Average daily gain (kg/day)  >150 days | |
| Female | Breeding herd replacement | 41 | 100 | 630*** | 0.82 | 0.67 | |
|  | Heifer sold after 1 year old without calving | 41 | 100 | 620 | 0.82 | 0.67 | |
| Male | Breeding herd replacement | 43 | 150 | 800 | 1.04 | 1.08 | |
|  | Young bull | 43 | 150 | 666 | 1.04 | 1.08 | |
| Veal production (dairy sector) | | | | | | | |
|  | | Weight at birth (kg) | Age at introduction in feedlot/export (days) | Age at maturity (days) | Weight at introduction in feedlot/export (kg) | Average daily gain (kg/day) birth-7 days | Average daily gain (kg/day) 7 days to sale |
| Female | Veal | 41 | 7 | 182 | 45 | 0.8 | 1.1 |
| Male | Veal | 43 | 7 | 182 | 48 | 1 | 1.1 |
|  | Export | 43 | 7 |  |  | 1 |  |

*age and weight at maturity of breeding females (cows) correspond to the age/weight at first calving.

**culling weight of beef cows: 750 kg

***culling weight of dairy cows: 700 kg

**References**

1. Réseau d'Elevage Charolais. Poids et prix de vente des animaux charolais en 2010. Paris: Réseaux d'élevages pour le conseil et la prospective. 2011.

2. Groupe Economie du Bétail Institut de l'Elevage. La production de viande bovine en France: qui produit quoi, comment et où? Paris: Institut de l'Elevage. 2011.

3. Bovins Croissance. Résultats 2012 des élevages bovins viande suivis par Bovins Croissance. Paris: Inosys Réseau d'Elevages, Bovins Croisssance, Institut de l'Elevage. 2013.

4. Institut de l'Elevage. Réseau Veau de Boucherie 2011-2013. Paris: Institut de l'Elevage. 2014.

5. Groupe Economie du Betail Institut de l'Elevage. Le dossier économie de l'Elevage - 2010 L'année économique viande bovine. Perspective 2011. Paris: Institut de l'Elevage. 2011.
