# Supplementary material for: Linking disease epidemiology and livestock productivity: The case of bovine respiratory disease in France
Source: PLoS One. 2017 Dec 5;12(12):e0189090. doi: 10.1371/journal.pone.0189090 (PMC5716546; doi:10.1371/journal.pone.0189090)
Supplement: S3 Table — (DOCX) [file pone.0189090.s006.docx]

**S3 Table. Fixed parameters used in the productivity model: breeding milk production performances and nutritional parameters.**

| Sector | Beef | Dairy |
| --- | --- | --- |
| Culling rate of the breeding herd | 21% | 27% |
| Parturition rate | 92% | 83% |
| Proportion birth of twins | 4% | 4% |
| Proportion females survive at 1 day | 96.8% | 94% |
| Proportion males survive at 1 day | 95.3% | 92.1% |
| Sex ratio of the breeding herd (male/female) | 4.1% | 4.1% |
| Weight loss at the beginning of lactation period | 0 | 35kg |
| Known quantity of milk offtake per lactation (kg) | 0* | 9215** |
| Milk protein concentration (g/kg) | 32.1 | 32.1 |
| Milk fat concentration (g/kg) | 39.7 | 39.7 |
| Metabolizability of milk (%) | 90 | 90 |
| Metabolizability of forage (%) | 55 | 55 |

*all the milk is considered to be suckled by farm calves

**all the milk is considered to be sold

**References**

1. Groupe Economie du Bétail Institut de l'Elevage. La production de viande bovine en France: qui produit quoi, comment et où? Paris: Institut de l'Elevage. 2011.

2. Bovins Croissance. Résultats 2012 des élevages bovins viande suivis par Bovins Croissance. Paris: Inosys Réseau d'Elevages, Bovins Croisssance, Institut de l'Elevage. 2013.

3. Douguet M. Résultats de Contrôle Laitier France 2011. Paris: Institut de l'Elevage. 2012.

4. Perrin JB, Ducrot C, Vinard JL, Hendrikx P, Calavas D. Analyse de la mortalité bovine en France de 2003 à 2009. INRA Prod Anim. 2011;24(3):235-44.
