# Supplementary material for: Linking disease epidemiology and livestock productivity: The case of bovine respiratory disease in France
Source: PLoS One. 2017 Dec 5;12(12):e0189090. doi: 10.1371/journal.pone.0189090 (PMC5716546; doi:10.1371/journal.pone.0189090)
Supplement: S4 Table — (DOCX) [file pone.0189090.s007.docx]

**S4 Table. Market prices used in the study (2015-2016 prices)**

| Product | Carcass quality* | | Price | Proportion of carcass categories | Reference |
| --- | --- | --- | --- | --- | --- |
| Beef young bull  (euro/kg live weight) | U | | 2.25 | 0.63 | 14, 20 |
|  | R | | 1.9 | 0.37 |  |
| Dairy young bull (euro/kg live weight) | O | | 1.5 | 0.67 |  |
|  | P | | 1.25 | 0.33 |  |
| Beef heifer  (euro/kg live weight) | U | | 2.7 | 0.28 |  |
|  | R | | 2 | 0.72 |  |
| Dairy heifer  (euro/kg live weight) | O | | 1.3 | 0.38 |  |
|  | P | | 1 | 0.62 |  |
| Veal  (euro/kg carcass) | O | | 5.8 | 0.94 | 14, 21 |
|  | P | | 5.1 | 0.06 |  |
| Carcass yield of veal calves (%) | | | 55 | | 20 |
| Female beef weanling  (euro/kg live weight)  (exported or slaughtered) | | | 2 | |  |
| Male beef light weanling  (euro/kg live weight)  (exported, slaughtered, or sold to feedlot) | | | 3 | |  |
| Male beef heavy weanling  (euro/kg live weight)  (exported or slaughtered) | | | 2.75 | |  |
| Male dairy sold to feedlot  (euro/kg live weight) | | | 1.5 | |  |
| Culled breeding cattle  (euro/ kg live weight) | | Beef | 1.85 | |  |
|  |  | Dairy | 0.95 | |  |
| 1 week old dairy calf (euro/head) | | | 100 | |  |
| Milk (euro/kg) | | | 0.3 | | 22 |
| Average veterinary cost of BRD cases (euro/case) | | | 24.2 | | 5 |

* According to the European carcass classification system, standard and downgraded carcasses of beef young bulls and heifers are ranked “U” and “R” respectively. Standard and downgraded carcasses of dairy young bulls, dairy heifers and veal calves are ranked “O” and “P” respectively.

**References**

1. Martineau C, Bertrand G, Kergoulay P. Indicateurs Zootechniques et Sanitaires. Veaux de boucherie. Le Rheu, France: Institut de l'Elevage, GIE Lait-Viande de Bretagne, la Chambre Régionale d'Agriculture de Bretagne. 2007.

2. Groupe Economie du Bétail Institut de l'Elevage. La production de viande bovine en France: qui produit quoi, comment et où? Paris: Institut de l'Elevage. 2011.

3. Web-Agri. Marchés de la Viande. Terre-Net Média; 2016. Available from: http://www.web-agri.fr/gros-bovins-boucherie/504.

4. FNICGV. Cotations Nationales - Veaux de Boucherie. Paris: Institut de l'Elevage. 2015.

5. Web-Agri. Cotation physique du lait. Terre-net Média; 2016. Available from: http://www.web-agri.fr/observatoire_marches/lait.html.
