# Supplementary material for: Linking disease epidemiology and livestock productivity: The case of bovine respiratory disease in France
Source: PLoS One. 2017 Dec 5;12(12):e0189090. doi: 10.1371/journal.pone.0189090 (PMC5716546; doi:10.1371/journal.pone.0189090)
Supplement: S5 Table — (DOCX) [file pone.0189090.s008.docx]

**S5 Table. Additional variable farming costs (per cattle-year)**

|  | Cattle population size (x1000 heads) [1]  (2010) | Number of full-time equivalent [2]  (2010) | Number of full-time equivalent per 1000 head-year  (2010) | Opportunity cost of labour (EURO/head-year)* | Other variable costs (including littering, water distribution and veterinary care)  (EURO/head-year) | Total additional costs  (EURO/head-year) |
| --- | --- | --- | --- | --- | --- | --- |
| Dairy | 7 741 | 115 157 | 14.88 | 268 | 195  [Ref: 3] | 463 |
| Beef | 11 319 | 103 792 | 9.17 | 165 | 77  [Ref:4] | 242 |
| Veal | 538 | 1 766 | 3.28 | 59 | 64  [Ref:5] | 123 |

*****Labour cost was calculated as: $L=\frac{CP}{N}$

With $C$ the opportunity cost of one equivalent full time employment, $P$ the number of equivalent full time workers employed by the considered sector and $N$ the cattle population size of the considered sector. Opportunity cost of one equivalent-full-time work per year in 2016: $C$ = 18 000 euro.

**References**

1. Agreste Conjoncture. Agreste Infos rapides - Animaux de boucherie- Bovins cheptel. Montreuil-Sous-Bois, France: Ministère de l'Agriculture, de l’Agroalimentaire et de la Forêt - Service de la Statistique et de la Prospective; 2013. Available from: http://www.agreste.agriculture.gouv.fr/conjoncture/animaux-de-boucherie/bovins/.

2. Lang A, Perrot C, Dupraz P, Tregaro Y, Rosner PM. GIS Elevages Demain - Les emplois liés à l’élevage français. Paris: Institut National de la Recherche Agronomique. 2015.

3. INOSYS - Réseaux d'Elevage. Repères techniques et économiques en élevage laitier - Résultats 2014. Paris: Institut de l'Elevage. 2015.

4. Réseaux d’élevage Auvergne L, Aveyron,. Références Systèmes Bovins Viande des Réseaux d'élevage Cantal, Lozère, Aveyron et Haute Loire. Aubière, France: Chambres d'agriculture - EDE - Institut de l'Elevage. 2013.

5. Chambres d'Agriculture de Bretagne. L’Observatoire Technico-économique Veaux de Boucherie. Rennes, France: Chambres d'Agriculture de Bretagne. 2014.
